# Role of AhR/ARNT system in skin homeostasis

**DOI:** 10.1007/s00403-014-1481-7

**Published:** 2014-06-26

**Authors:** Masutaka Furue, Masakazu Takahara, Takeshi Nakahara, Hiroshi Uchi

**Affiliations:** 1 Department of Dermatology, Kyushu University, Maidashi 3-1-1, Higashiku, Fukuoka, 812-8582 Japan; 2 Research and Clinical Center for Yusho and Dioxin, Kyushu University, Fukuoka, Japan; 3 Division of Skin Surface Sensing, Kyushu University, Fukuoka, Japan

**Keywords:** Aryl hydrocarbon receptor, Aryl hydrocarbon receptor nuclear translocator, Dioxin, *Malassezia*, Melanogenesis, Th17, Treg, Ultraviolet

## Abstract

Aryl hydrocarbon receptor (AhR) is a ligand-dependent transcription factor that binds to structurally diverse synthetic and naturally occurring chemicals including dioxins, flavonoids, tryptophan photoproducts, and *Malassezia* metabolites. Upon binding to its ligands, cytoplasmic AhR translocates to the nucleus, heterodimerizes with aryl hydrocarbon receptor nuclear translocator (ARNT), and mediates numerous biological and toxicological effects by inducing the transcription of various AhR-responsive genes. AhR ligation controls oxidation/antioxidation, epidermal barrier function, photo-induced response, melanogenesis, and innate immunity. This review summarizes recent advances in the understanding of the regulatory mechanisms of skin homeostasis mediated by the AhR/ARNT system.

## Introduction

The skin is a highly sophisticated sensory organ covering the surface of the body. The sensing of external physiological and chemical stimuli plays key roles in self-defense and homeostasis. Aryl hydrocarbon receptor (AhR, also called dioxin receptor) is a chemical receptor that responds to exogenous and endogenous chemicals by inducing/repressing the expression of several genes with toxic or protective effects in a wide range of species and tissues [8]. The best-characterized high-affinity ligands for AhR include several ubiquitous hydrophobic environmental contaminants, such as halogenated and nonhalogenated polycyclic aromatic hydrocarbons (e.g., dioxins and benzo[a]pyrene) [8, 26]. Recent studies have also demonstrated that AhR can bind and be activated by structurally diverse chemicals, such as various phytochemicals [34, 52], *Malassezia* metabolites [45], and photo-induced chemicals [9, 93, 94] with a wide range of affinities. As keratinocytes, sebocytes, fibroblasts, melanocytes, endothelial cells, Langerhans cells, and other immune cells possess AhR [23, 30, 31, 34, 82, 84], the physiological and pathological processes of skin homeostasis and differentiation are variably affected by the ligand-dependent activation of the AhR signal transduction pathway.

## AhR/ARNT signaling

**Fig. 1:**
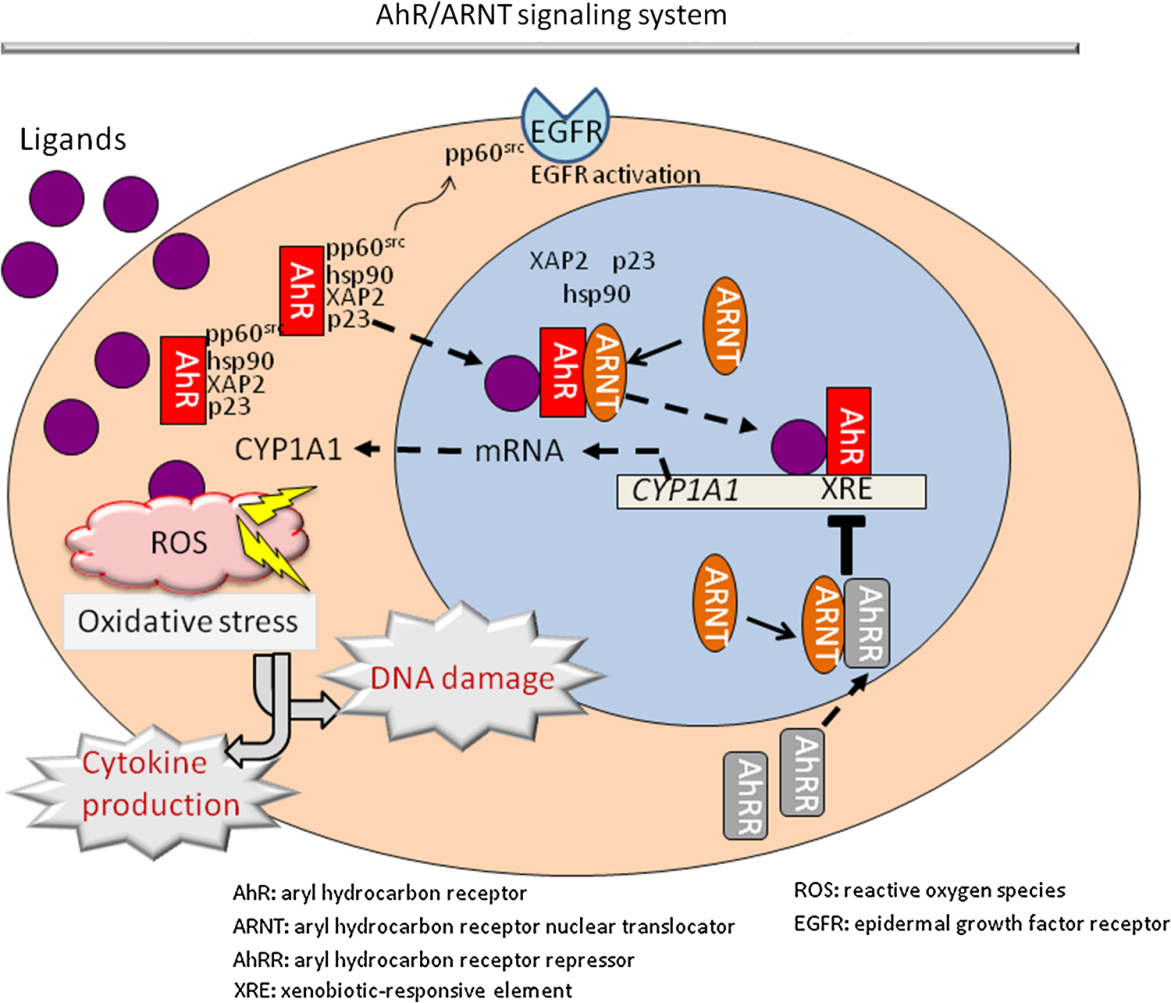
Schematic representation of the AhR/ARNT signaling system. Aryl hydrocarbon receptor (AhR) resides in the cytoplasm as a protein complex with hsp90, XAP2, and p23. Various external and internal ligands like dioxins, dietary flavonoids, *Malassezia* metabolites, and ultraviolet light-induced metabolites bind to and activate AhR. Upon ligand binding, ligand–AhR protein complex translocates into the nucleus, where AhR nuclear translocator (ARNT) binds to it, releasing hsp90, XAP2, p23, and pp60^src^. The ligand–AhR–ARNT complex binds to the xenobiotic-responsive element (XRE) and induces the transcription of responsive genes such as *cyp1A1*. During the process of metabolism of ligands (e.g., dioxins) by CYP1A1, a large number of reactive oxygen species (ROS) are produced. This ROS generation is closely related to various cellular responses, such as cytokine production and DNA damage. Meanwhile, the dissociated pp60^src^ activates epidermal growth factor receptor (EGFR) and induces its internalization and nuclear translocation. Moreover, the AhR signaling induces the transcription of AhR repressor (AhRR). This induced AhRR forms a heterodimer with ARNT, which competes with AhR/ARNT heterodimer to bind to the XRE sequence, consequently inhibiting AhR transcriptional activity

Aryl hydrocarbon receptor is a basic helix-loop-helix/Per-ARNT-Sim (bHLH-PAS)-containing transcription factor essential for adaptive responses to xenobiotics by inducing xenobiotic-metabolizing enzymes such as cytochrome P450 1A1 (CYP1A1) [51]. Most AhR ligands such as 2,3,7,8-tetrachlorodibenzo-*p*-dioxin (TCDD) and benzo[a]pyrene are very hydrophobic; these ligands enter target cells via diffusion and bind to cytosolic AhR, which exists in an inactive or latent state as a multiprotein complex containing heat shock protein 90 (hsp90), immunophilin-like XAP2, the co-chaperone protein p23, and pp60^src^ (Fig. 1) [1]. Upon ligand binding, AhR is presumed to undergo a conformational change that exposes its N-terminal nuclear localization sequence, facilitating the nuclear translocation of the AhR–ligand complex. The translocated HSP90-bound AhR subsequently dissociates from the HSP90 complex by binding to a structurally related nuclear protein, aryl hydrocarbon receptor nuclear translocator (ARNT) [22]. AhR–ARNT dimerization facilitates the conversion and transformation of the ligand–AhR–ARNT complex into its high-affinity DNA-binding form [22, 75]. Meanwhile, the dissociated pp60^src^ activates epidermal growth factor receptor (EGFR) and induces the internalization and nuclear translocation of EGFR (Fig. 1) [1, 39].

The binding of the heterodimeric ligand–AhR–ARNT complex to its specific DNA recognition site, namely, the xenobiotic-responsive element (XRE) or dioxin-responsive element, upregulates the transcription of responsive genes such as *cyp1a1*. CYP1A1 is a member of a multigene family of xenobiotic-metabolizing enzymes [8, 26, 51]. Besides its physiological role in the detoxification of polycyclic aromatic compounds, the activity of this enzyme can be deleterious because it generates mutagenic metabolites and reactive oxygen species (ROS). In addition to the upregulation of CYP1A1 and consequent ROS production, a high-affinity AhR ligand, TCDD, causes a broad spectrum of biochemical and toxicological effects, such as teratogenesis, immunosuppression due to thymic involution, and tumor promotion. Extensive studies on the function of AhR using AhR-deficient mice have demonstrated that AhR is responsible for most, if not all, of the toxic effects caused by TCDD [51].

Moreover, the AhR signaling pathway is downregulated via feedback inhibition, particularly via the activity of the AhR repressor (AhRR) [50, 51]. The AhRR promoter has a functional XRE sequence, and its gene expression is enhanced upon the ligand activation of AhR. The induced AhRR forms a heterodimer with ARNT, which competes with AhR/ARNT heterodimer to bind to the XRE sequence, consequently inhibiting AhR transcriptional activity. However, the feedback competition of AhRR/ARNT in AhR/ARNT signaling is a complicated and poorly understood regulatory system (Fig. 1) [51].

## Role of AhR/ARNT in oxidative stress

The cells lining the outer and inner surfaces of the body, such as keratinocytes and airway epithelial cells, express AhR/ARNT complex [4, 84]. Benzo[a]pyrene actively induces the nuclear translocation of AhR and subsequent CYP1A1 and ROS generation, leading to DNA damage (i.e., 8-hydroxydeoxyguanosine production) and interleukin 8 (IL-8) production in keratinocytes, as well as mucin (MUC5AC) production in airway epithelial cells [4, 84]. AhR knockdown by specific siRNA abrogates this series of reactions, indicating their dependence on AhR. These results are concordant with the fact that the carcinogenic and inflammatogenic activities of benzo[a]pyrene and TCDD are abolished in AhR-null mice [51, 73]. Using an ex vivo skin organ culture system, Costa et al. [7] confirmed that benzo[a]pyrene actually upregulates CYP1A1 expression, ROS production, and subsequent protein peroxidation. As benzo[a]pyrene is one of the major harmful ingredients of tobacco smoke, AhR-mediated IL-8 production may explain why tobacco smoking exacerbates IL-8-related inflammatory skin diseases such as psoriasis and palmoplantar pustulosis [2, 10, 84].

**Fig. 2:**
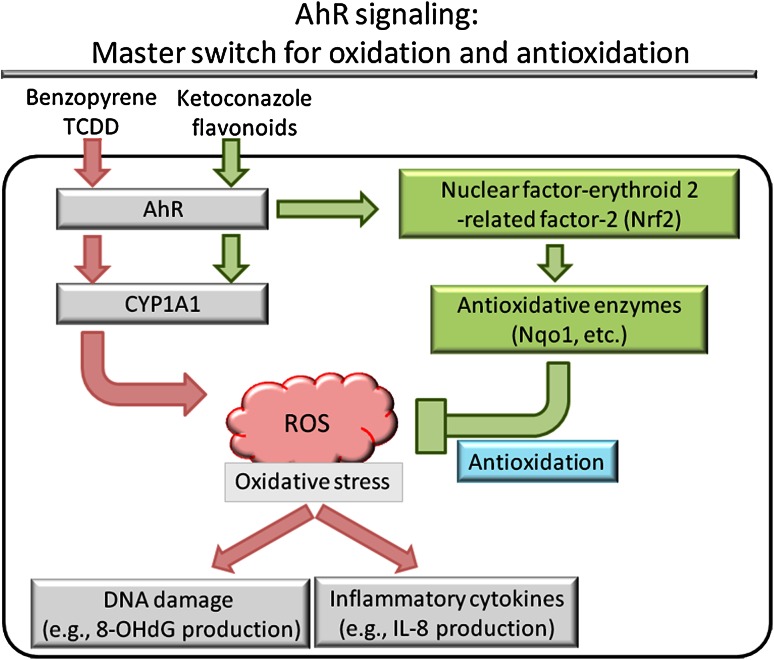
AhR ligation induces not only oxidative stress but also antioxidative response in a ligand-dependent manner. Environmental pollutants such as benzo[a]pyrene and TCDD bind to AhR and induce ROS production, DNA damage, and inflammatory cytokine production. In contrast, ketoconazole and certain flavonoids bind to AhR, resulting in the activation of Nrf2 and subsequent induction of antioxidative enzymes such as Nqo1. These antioxidative enzymes inhibit ROS production, DNA damage, and inflammatory cytokine production. Thus, AhR acts as a master switch for oxidation and antioxidation

In addition to oxidative stress, recent studies have demonstrated that the AhR/ARNT system mediates antioxidative and protective signaling in response to different ligands, such as flavonoids, herbal medicines, and azoles (Fig. 2) [20, 21, 28, 59, 83]. For example, ketoconazole binds and induces the nuclear translocation of AhR without producing ROS. Instead, it activates nuclear factor-erythroid 2-related factor-2 (Nrf2) and subsequently NAD(P)H:quinone oxidoreductase 1 (Nqo1), which are key molecules that protect cells from ROS-induced oxidative damage [20, 28, 83]. Ketoconazole actually inhibits benzo[a]pyrene- and tumor necrosis factor-alpha (TNF-α)-induced ROS and IL-8 production, which is abolished by AhR or Nrf2 knockdown, but not by AhRR knockdown [83]. Similar findings have been obtained with traditional herbal remedies such as *Bidens pilosa* extract [34]. Both benzo[a]pyrene and TNF-α also induce marked ROS production in endothelial cells. *B. pilosa* extract potently inhibits ROS production by upregulating Nrf2 and Nqo1, which are abrogated by knockdown of AhR or Nrf2 [34]. The tea flavonoid epigallocatechin gallate upregulates Nrf2 and Nqo1 expression while downregulating AhR and CYP1A1 expression [21]. Quercetin, one of the flavonoids, efficiently induces AhR activation and CYP1A1 production [53]. However, it potently inhibits ultraviolet B (UVB)-induced ROS production [95]. In addition, quercetin also induces AhRR mRNA upregulation [59]. Benzo[a]pyrene-induced ROS production is AhR-dependent since it is inhibited by siRNA specific for AhR [84]; however, ketoconazole- and quercetin-mediated AhR activation occurs without ROS production [83, 95]. Therefore, the AhR-related production of ROS is likely to be evoked in a ligand-dependent manner. These complicated results indicate that the AhR/ARNT system acts as a master switch for up and downregulating oxidative stress by modulating diverse genes (e.g., those of AhR, AhRR, CYP1A1, Nrf2, and Nqo1). However, the precise mechanisms by which various phytochemicals and environmental pollutants differentially affect the AhR/ARNT system remain largely unknown.

## Role of AhR/ARNT in epidermal barrier function

Coal tar comprises at least 10,000 high-molecular-weight hydrocarbon and aromatic compounds, which may target the AhR/ARNT system. Topical coal tar remedies have been widely used to treat inflammatory skin diseases for at least two millennia [49]. Using organotypic skin models with primary keratinocytes from atopic dermatitis patients and controls, van den Bogaard et al. [86] demonstrated that coal tar activates AhR, resulting in the induction of epidermal differentiation (i.e., upregulation of filaggrin, loricrin, and hornerin expression), and thickens the cornified layer. Furthermore, AhR knockdown by siRNA completely abrogates this effect. In atopic dermatitis patients, coal tar completely restores the expression of major skin barrier proteins including filaggrin. Coal tar also diminishes spongiosis, apoptosis, and CCL26 expression in organotypic skin stimulated by the Th2 cytokines IL-4 and IL-13 via the dephosphorylation of STAT6; this is most likely due to the AhR-regulated activation of the Nrf2 antioxidation pathway [86]. Many studies have shown that AhR mediates the upregulation of epidermal differentiation [42, 43, 67, 77]. TCDD increases the quantity of cornified envelopes in monolayer cultures and organotypic cultures of keratinocytes [43]. TCDD also enhances filaggrin, involucrin, transglutaminase, and IL-1β expression [42, 63, 77]. In addition, TCDD exposure significantly augments the mRNA expression of other epidermal differentiation complex genes [38]: repetin, hornerin, late cornified envelope (LCE) 3E, LCE3A, LCE2B, LCE2A, LCE1C, small proline-rich protein (SPRR) 1A, SPRR2A, SPRR2B, S100A9, S100A12, and S100A7 [77]. Accordingly, the targeted ablation of ARNT in mouse epidermis results in profound defects in desquamation and epidermal barrier function, particularly decreased filaggrin and SPRR2A expression [18]. It is quite interesting that the increase in cornified envelope proteins such as SPRRs decrease oxidative stress by quenching excess ROS [88, 89]. Recent work by Kennedy et al. [33] has also revealed that TCDD increases the expression of 40 % of the genes of the epidermal differentiation complex found on chromosome 1q21, such as hornerin, filaggrin, SPRR2B, SPRR4, and LCE3A. In addition, TCDD increases the expression of 75 % of the genes required for de novo ceramide biosynthesis, leading to the overproduction of ceramides 1, 2, 3, 4, 5, 6, 7, and 9 without affecting the levels of cholesterol and free fatty acids. Moreover, the cornified envelope formation induced by TCDD is blocked in the presence of antioxidative agents, quercetin, catalase, or *N*-acetyl-l-cysteine, indicating an important role for ROS production in the TCDD-induced acceleration of epidermal terminal differentiation [33].

**Fig. 3:**
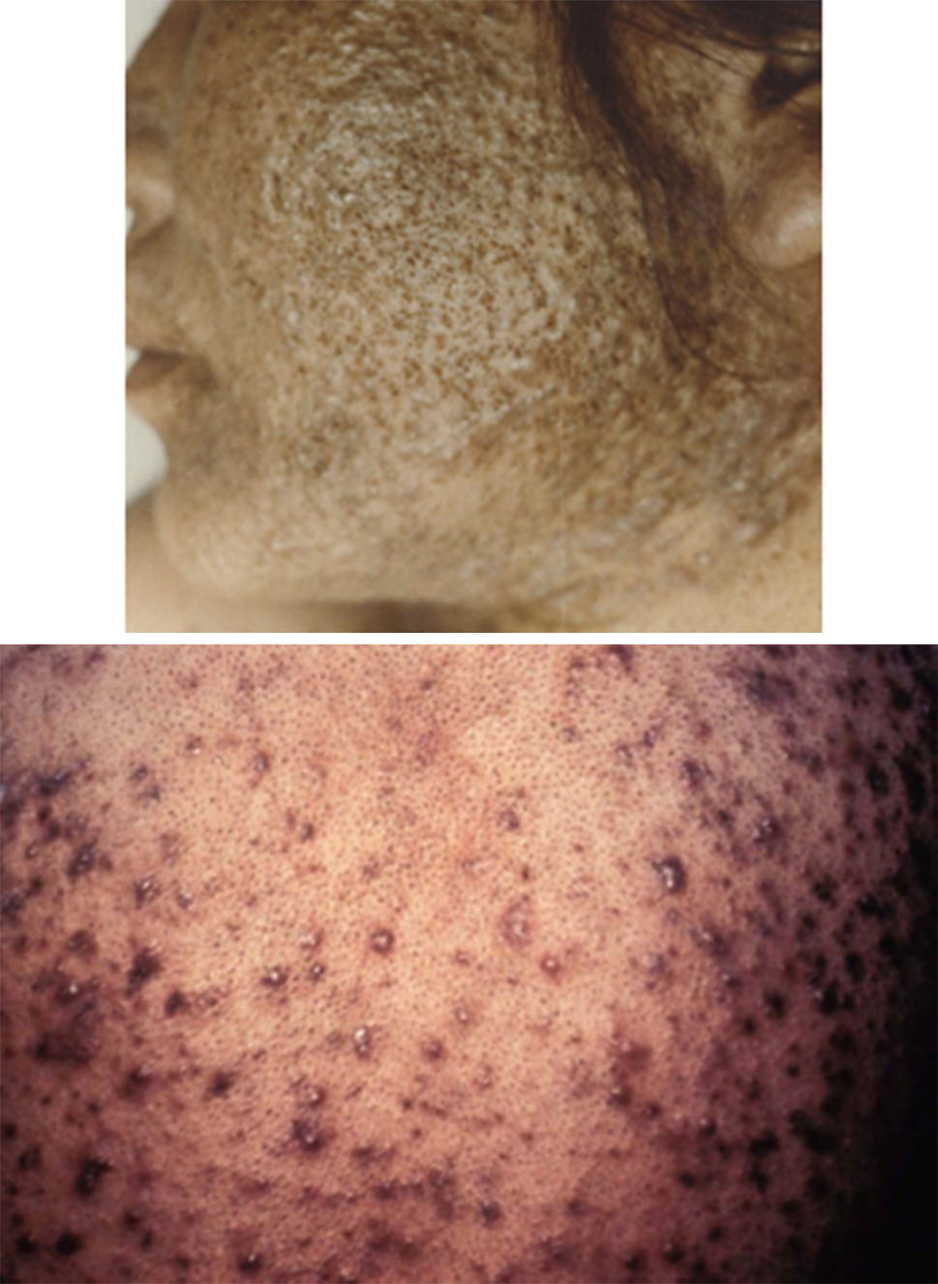
Severe chloracne in Yusho patients (oral intoxication of a high concentration of 2,3,4,7,8-pentachlorodibenzofuran)

Exposure to extremely high concentrations of dioxins induces chloracne in humans, as was demonstrated in the Yusho and Seveso industrial accidents in Japan and Italy, respectively (Fig. 3) [3, 14]. The pathology of chloracne is characterized by hyperkeratinization of the interfollicular squamous epithelium, hyperproliferation and hyperkeratinization of hair follicle cells, and a metaplastic response of the sebaceous glands [29, 61, 63, 68, 76, 85]. Highly lipophilic dioxins appear to accumulate in and are excreted via sebaceous glands and sebum [25, 74], which may efficiently excrete dioxins from the intoxicated body [47]. TCDD also affects the differentiation of sebaceous gland cells, probably by switching human sebocytes toward keratinocyte-like differentiation [29]. Although the precise mechanism behind chloracne is not understood, sustained AhR hyperactivation and exaggerated hyperkeratinization of pilosebaceous units may be the cause of this devastating toxicity.

AhR-null mice appear normal at birth, but their growth is slightly slower than that of wild-type mice during the first few weeks of life. Thereafter, they catch up and no difference is apparent in animals over 12 weeks of age [73]. Tauchi et al. [80] generated transgenic mice expressing the constitutively active form of AhR in keratinocytes. At birth, these transgenic mice were normal, but severe skin lesions developed postnatally, along with marked scratching. Prominent epidermal acanthosis and hyperkeratosis were evident, with severe dermal infiltration of lymphocytes and polymorphonuclear cells in the lesional skin. Th2-skewed immune deviation was evident in the skin lesions and spleen in the transgenic mice, with an elevated circulating IgE level [80]. Interestingly, keratinocyte-specific *ARNT*-deficient mice generated by a *K5*-*Cre*-*loxP* system exhibit severe skin barrier dysfunction and die because of rapid dehydration [79]. The keratinocyte-specific *ARNT* disruption results in significant changes in the amount and composition of ceramides, but not cholesterol and fatty acids. The most prominent changes in the ceramide composition of the *ARNT* -null epidermis are observed for ceramide 2 and ceramide 5. The 4-sphingenine that these ceramides normally contain is largely replaced by sphinganine due to the impaired transcription of dihydroceramide desaturase isozyme, *Des*-*2* [79]. Another type of epidermal *ARNT* -null mice generated by Geng et al. using a *K14*-*Cre*-*loxP* system also exhibits the upregulation of genes of the epidermal differentiation complex (S100A8, S100A9, S100A10, SPRR1, and SPRR 2) and the alteration of ceramides. In addition, the *ARNT*-null epidermis exhibits the upregulation of secretory leukocyte protease inhibitor (Slpi), which inhibits stratum corneum chymotryptic enzyme (kallikrein 7), leading to hyperkeratosis due to impaired corneodesmosome degradation and delayed detachment of corneocytes [18]. These studies highlight the importance of the AhR/ARNT system in keratinocyte terminal differentiation.

Although ROS production has been shown to be a prerequisite in the TCDD-induced upregulation of keratinocyte terminal differentiation [33], excessive antioxidant activity also hampers the epidermal barrier function [69, 71]. *K5*-*Cre*-*Nrf2* transgenic mice generated by Schäfer et al. [69] express high levels of constitutively active Nrf2 in the epidermis together with the overexpression of Nqo1 and other antioxidative enzymes. Unexpectedly, their skin is dry with hair loss and scaling. In terms of their histology, epidermal acanthosis and hyperkeratosis are evident, with sebaceous gland enlargement and hair follicle abnormality. Like ARNT-null keratinocytes [18], the Nrf2-transgenic keratinocytes exhibit the upregulated expression of Slpi, SPRR2d, and SPRR2 h. The upregulated Slpi again inhibits kallikrein 7 activity, leading to impaired detachment of corneocytes, which results in hyperkeratosis [69]. The prolonged Nrf2 activation in *K5*-*Cre*-*Nrf2* transgenic mice also causes sebaceous gland enlargement and seborrhea due to upregulation of epigen, a recently identified ligand for EGFR. Upon aging, the upregulation of Slpi, SPRR2d, and epigen in the pilosebaceous unit results in infundibular acanthosis, hyperkeratosis, and cyst formation mimicking chloracne [71]. Therefore, Slpi, SPRR2d, and epigen are crucial target molecules of Nrf2 in modifying epidermal and pilosebaceous differentiation. Kelch-like ECH-associated protein-1 (Keap1) is an Nrf2 repressor protein. In keeping with the findings in Nrf2-transgenic mice, Keap1-null mutation induces constitutive Nrf2 activation leading to hyperkeratosis [90]. With regard to the effect of EGFR signaling on the AhR/ARNT system, EGFR signaling blocks TCDD-induced CYP1A1 production as well as filaggrin upregulation [78]. Meanwhile, EGFR signaling is capable of activating the Nrf2/Nqo1 system [62]. However, there remain a plethora of unanswered questions in terms of the crosstalk among AhR, Nrf2, and EGFR signaling.

## Role of AhR/ARNT in photobiology, melanogenesis, and immunodermatology

The critical roles of ROS and the AhR/ARNT system in photobiology have been elegantly reviewed by Schäfer et al. [70] and Krutmann et al. [37]. Besides environmental contaminants and dietary constituents [52, 57], many endogenous compounds including various indoles, heme, and arachidonic acid metabolites are AhR agonists; moreover, tryptophan is the precursor of many of the most active ligands for AhR [57]. Fritsche et al. [12] first demonstrated that UVB irradiation induces the intracellular tryptophan photoproduct, 6-formylindolo[3,2-*b*]carbazole (FICZ), which eventually induces the nuclear translocation of AhR. Furthermore, AhR-knockout mice exhibit compromised UVB responsiveness. Thus, AhR signaling is an integral part of the UVB stress response [12]. FICZ, which is formed upon the exposure of tryptophan solutions, cell culture media, or cells to UV radiation, binds to AhR with greater affinity than TCDD [57]. UVB is the most efficient means of generating FICZ from tryptophan [57, 60]. FICZ formation increases 40- to 400-fold in the presence of the photosensitizer riboflavin (vitamin B_2_), especially upon exposure to UVA and visible light; this is because riboflavin absorbs light efficiently at these longer wavelengths [57, 60]. This relatively easy conversion of tryptophan to FICZ in the presence of riboflavin and light suggests that the same process could occur in the skin. Thus, the formation of FICZ may explain the reported UV-dependent activation of CYP1 enzymes in human skin [93]. Indeed, FICZ metabolites are detected in human urine [93].

6-Formylindolo[3,2-*b*]carbazole is a high-affinity ligand for AhR; its *K*_*d*_ value is 0.07 nM, which is two or more orders of magnitude less than that of low-affinity ligands such as prostaglandin and lipoxin derivatives [56, 57, 60]. FICZ upregulates the expression of AhR-responsive genes (e.g., CYP1A1) in an efficient but transient manner; this is because FICZ is rapidly metabolized by CYP1A1 in a feedback mechanism [92–94]. This sequence of events, which is typical of autoregulatory loops in biological signaling, suggests that FICZ may be an important physiological ligand for AhR. UVB as well as FICZ does indeed activate the AhR/ARNT system and upregulates the gene expression of CYP1A1 and matrix metalloproteinase 1, which are abolished in the presence of AhR antagonists [81]. Both FICZ and UVB activate EGFR and its downstream signaling extracellular signal-regulated kinases 1 and 2 and cyclooxygenase-2 (COX-2) [1, 12]. The induction of CYP1A1 and COX-2 mRNAs in the skin of mice exposed to UVB was blunted in AhR-deficient mice [12]. Moreover, it has been shown that AhR signaling may play an antiapoptotic role in UVB-exposed skin [11], strongly indicating that AhR signaling does in fact contribute to photocarcinogenesis, as proposed by Agostinis et al. [1]. However, we have to keep in mind that FICZ is a tiny fraction of tryptophan photoproducts and the outline proposed above has yet to be confirmed empirically. The role of FICZ in cutaneous photobiology remains largely unclear.

Normal murine melanocytes also express functional AhR [31]. Using standard UVB tanning protocols, Jux et al. [31] have demonstrated that AhR-deficient mice develop a significantly weaker tan than wild-type mice and that epidermal tyrosinase activity is decreased in AhR-deficient mice. However, tanning response and tyrosinase activity are normal in keratinocyte-specific AhR-conditional knockout mice, indicating that downregulation of the melanogenic response is a direct effect of UVB/AhR signaling on melanocytes [31]. In fact, AhR can modulate melanogenesis by controlling the expression of melanogenic genes in melanocytes. Luecke et al. have reported that exposing normal human melanocytes to TCDD activates the AhR signaling pathway, as well as the AhR-dependent induction of tyrosinase activity, with the elevation of total melanin content. Neither the induction of tyrosinase enzyme activity nor that of total melanin could be attributed to the enhanced cell proliferation of melanocytes; instead, they are due to the induction of tyrosinase and tyrosinase-related protein 2 gene expression [44]. Nakamura et al. [54] have demonstrated that tobacco smoke extract exerts similar melanogenic effects by activating AhR in melanocytes. In this context, Schallreuter et al. have demonstrated that AhR signaling is severely impaired in the lesional and nonlesional skin in cases of vitiligo, despite the presence of FICZ [72]. UVB phototherapy is the mainstay treatment for vitiligo. A recent study by Lan et al. [39] has revealed that excimer light (peak wavelength 308 nm) is more potent at inducing melanogenesis of cultured melanocytes than narrow-band UVB (peak wavelength 311 nm) because the former upregulates AhR–EGFR-dependent tyrosinase activity more efficiently than the latter.

Wang et al. [91] examined the functional AhR gene polymorphisms and suggested that the T allele of rs10249788, which is located in the promoter of the AhR gene, is associated with a protective effect against vitiligo in Han Chinese populations. Concordantly, our recent study clarified that a transcription factor, nuclear factor 1-C (NF1C), which suppresses AhR gene transcription, preferentially binds to the C allele over the T allele at rs10249788 [41]. Therefore, it is conceivable that subjects with the T allele at rs10249788 express higher levels of AhR and are more melanogenic than those with the C allele.

**Fig. 4:**
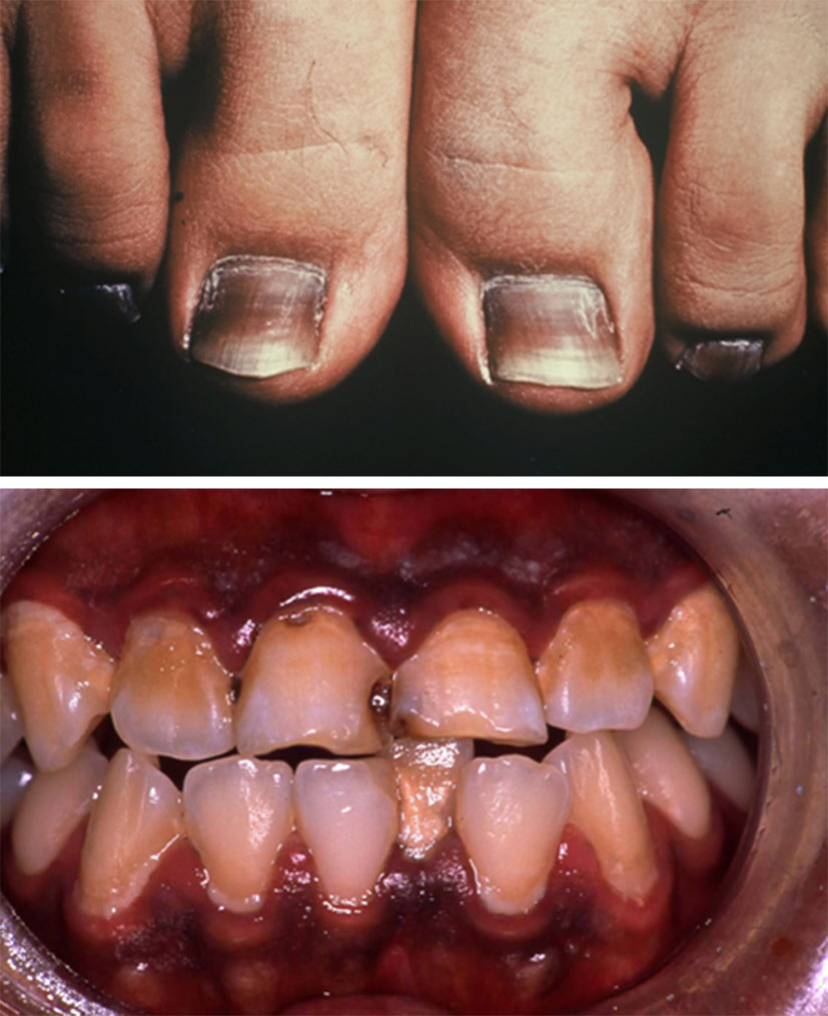
Hyperpigmentation in Yusho patients

AhR-mediated melanogenesis may also explain the marked hyperpigmentation that occurred in victims of the Yusho industrial accident, who were exposed to extremely high levels of dioxin-related compounds (Fig. 4) [27, 53]. However, it should be mentioned that skin pigmentation was not always recognized in the victims of TCDD intoxication [19, 68]. Yusho patients may be different, as they were exposed to a wide variety of polyhalogenated polycyclic hydrocarbons, in particular polychlorinated biphenyls, which may simply produce a charcoal-like blackish skin color.

*Malassezia* yeasts are unique in that they are virtually the sole eukaryote among the microbial flora of the skin [15]. *Malassezia furfur* produces malassezin and other indole derivatives by converting l-tryptophan in culture medium [35, 48]. Since l-tryptophan is present in sweat [24], *Malassezia* is expected to produce these compounds on the surface of human skin in vivo. Indeed, human skin extracts from seborrheic dermatitis lesions infected by *Malassezia* contain indirubin, FICZ, indolo[3,2-b]carbazole (ICZ), and malassezin [16, 17]. Interestingly, these molecules are active AhR agonists and induce CYP1A1 production [45]. In the presence of malassezin, human melanocytes undergo apoptosis; this may explain why pityriasis versicolor induces depigmented macules [36]. Thus, how AhR discriminates similar but oppositely agonistic ligations by TCDD (i.e., in melanogenesis) and malassezin (i.e., in melanocyte apoptosis) is an intriguing issue [36, 54].

In the immune system, AhR appears to play a crucial role in T-helper 17 (Th17) and regulatory T (Treg) cells [13, 46, 64–66, 87]. AhR-deficient mice can develop Th17 cells, but fail to respond to AhR ligands to enhance Th17 cell development [87]. AhR activation during the induction of experimental autoimmune encephalomyelitis accelerates the onset and increases the pathology of this condition in wild-type mice, but not in AhR-deficient mice [87]. The development of Treg cells is reciprocally related to that of Th17 cells. The agonistic ligation of AhR by TCDD induces functional Treg cells, suppressing experimental autoimmune encephalomyelitis. On the other hand, AhR activation by FICZ interferes with Treg development, boosts Th17 cell differentiation, and increases the severity of experimental autoimmune encephalomyelitis in mice [65].

Aryl hydrocarbon receptor-mediated Treg cell induction appears to operate in UV-induced immunosuppression. Navid et al. [55] have demonstrated that the AhR antagonist 3-methoxy-4-nitroflavone reduces UV-mediated immunosuppression as well as the induction of Treg cells in murine contact hypersensitivity. Conversely, AhR activation by the agonist 4-*n*-nonylphenol suppresses the induction of contact hypersensitivity and induces antigen-specific Treg cells similarly to UV radiation. This has been further confirmed in AhR-knockout mice, which exhibit significantly reduced UV radiation- and 4-*n*-nonylphenol-induced immunosuppression [55].

Kynurenine is an important immunoinhibitory metabolite of tryptophan and is generated by indoleamine 2,3-dioxygenase (IDO) [58]. AhR activation by TCDD is required to induce IDO expression in dendritic cells. In the presence of lipopolysaccharide or CpG, bone marrow-derived dendritic cells skew the differentiation of naïve T cells toward Treg cells rather than Th17 cells, however, the capacity of developing Treg cells is deteriorated in AhR-deficient dendritic cells. The restoration of the Treg-inducible function in AhR-deficient dendritic cells by exogenous kynurenine indicates that AhR/IDO/kynurenine induction is important for the generation of tolerogenic dendritic cells under lipopolysaccharide or CpG stimulation [58].

Epidermal Langerhans cells also express AhR [30]. When cultured, AhR-deficient Langerhans cells show insufficient maturation and decreased expression of costimulatory molecules such as CD40, CD80, and CD24a. In keeping with this notion, AhR-deficient mice exhibit significantly weaker contact hypersensitivity to hapten [30]. The maturation of Langerhans cells is stimulated by granulocyte–macrophage colony-stimulating factor produced from surrounding keratinocytes. However, this production of granulocyte–macrophage colony-stimulating factor is significantly decreased in AhR-deficient keratinocytes [30]. Another immunocompetent bone marrow-derived cell type in the murine epidermis is the γδ T cells (dendritic epidermal T cells; DETCs) [32]. The proliferation and distribution of DETCs are markedly impaired in AhR-null mice due to insufficient expression of c-Kit, which is a downstream target molecule of AhR [32].

## Other studies

The AhR/ARNT system plays diverse and complex roles in tissue differentiation, immunoregulation, and carcinogenesis. As this system is fundamentally involved in various cellular responses, numerous papers have recently been published on it. The AhR/ARNT system is also important for the connection between food and health [40]. Exposure to AhR ligands through the human diet, even in the first weeks of life, is critical for the development of immune responses, as they control the maturation of innate lymphoid cells. Innate lymphoid cells drive immune responses against intestinal infections, and their generation is impaired in AhR-deficient mice. Mice fed a diet lacking natural AhR ligands suffer from deficient innate lymphoid cell generation and are prone to intestinal infection. The sole addition of the natural AhR ligand indole-3-carbinol to the diet restores both the generation of innate lymphoid cells and the immune response in an AhR-dependent manner. This highlights the importance of exposure to AhR agonists through the diet and their role in the maintenance of intestinal homeostasis [40]. Human blood actually contains a number of AhR ligands, such as resveratrol and indole-3-carbinol derived from vegetables, fruit, nuts, and herbs [5]. The complexity of regulatory mechanisms associated with the AhR/ARNT system has also been highlighted by a melanoma study by Contador-Troca et al. [6]; they found that AhR contributes to tumor-stroma interaction, that is, blocking melanoma growth and metastasis when expressed in tumor cells, but supporting melanoma when expressed in the stroma. Studies on skin surface sensing by AhR/ARNT should elucidate enigmatic host-environment interactions and may provide novel strategies for drug development.
